# Mechanism of p27 Unfolding for CDK2 Reactivation

**DOI:** 10.1038/srep26450

**Published:** 2016-05-23

**Authors:** Soumya Lipsa Rath, Sanjib Senapati

**Affiliations:** 1 Computational Biophysics Group, Bhupat and Jyoti Mehta School of Biosciences and Department of Biotechnology, Indian Institute of Technology Madras, Chennai, Tamil Nadu, India

## Abstract

Cell-cycle regulatory protein, CDK2 is active when bound to its complementary partner protein, CyclinA or E. Recent discovery of the Kip/Cip family of proteins has indicated that the activity of CDK2 is also regulated by a member protein, p27. Although, the mechanism of CDK2 inhibition by p27 binding is known from crystal structure, little is known about the mechanism of CDK2 reactivation. Here, we execute classical and accelerated molecular dynamics simulations of unphosphorylated- and phosphorylated-p27 bound CDK2/CyclinA to unravel the CDK2 reactivation mechanism at molecular-to-atomic detail. Results suggest that the phosphorylation of p27 Y88 residue (pY88-p27) first disrupts the p27/CDK2 hybrid β-sheet and subsequently ejects the p27 3_10_ helix from CDK2 catalytic cleft. The unbinding of p27 from CDK2/CyclinA complex, thus, follows a two-step unfolding mechanism, where the 3_10_ helix ejection constitutes the rate-limiting step. Interestingly, the unfolding of p27 leaves CDK2/CyclinA in an active state, where the prerequisite CDK2-CyclinA interfacial contacts were regained and ATP achieved its native position for smooth transfer of phosphate. Our findings match very well with NMR chemical shift data that indicated the flip-out of p27 3_10_ helix from CDK2 pocket and kinetic experiments that exhibited significant kinase activity of CDK2 when saturated with pY88-p27.

Cyclin Dependent Kinase 2 (CDK2) is a member of the eukaryotic Serine/Threonine protein kinase family that plays a key role in cell division cycle. CDK2 participates in cell cycle regulation at the G1/S boundary and assists the γ-phosphate transfer from ATP to peptide substrates belonging to downstream targets^1^,^2^. The activation of CDK2 involves two major steps: (i) its association with the regulatory subunit – CyclinA or CyclinE and (ii) phosphorylation of the residue Thr160^3^,^4^,^5^. Recent discovery of the Kip/Cip and INK4 family of proteins has indicated that the activity of CDKs is also regulated by this family of proteins, which bind to and inhibit the catalytic activity of CDKs for cell-cycle control^5^,^6^. One such inhibitor, p27 that belongs to Kip/Cip family, potently inhibits CDK2 kinase activity in G0 and early G1 phase and leads to cell-cycle arrest^7^. As cells enter the S phase, the progressive decrease in p27 level allows phospho-CDK2/CyclinA (pCDK2/CyclinA) and phospho-CDK2/Cyclin E (pCDK2/Cyclin E) complexes to regain the activity^5^,^8^.

In recent years, enormous effort has been put forward to understand the CDK2-inhibitory mechanism of p27 and also the mechanism of CDK2 reactivation^8^,^9^,^10^,^11^,^12^,^13^. The crystal structure of p27 bound pCDK2/CyclinA has provided a great deal of information about the mechanism of CDK2 inhibition by p27^11^. The structure revealed large conformational changes in and around the catalytic cleft of CDK2 due to strong association with p27, which effectively blocks ATP binding. The exploration of the mechanism of CDK2 reactivation, however, received momentum only very recently. Kinetic studies and NMR data suggested that the CDK2 reactivation mechanism involves the phosphorylation of certain p27 tyrosine residues by other tyrosine kinases^12^,^13^.

The crystal structure of pCDK2/CyclinA bound to the kinase inhibitory domain of p27 was solved at 2.3 Å resolution (Fig. 1, from here referred as p27/CDK2/CyclinA)^11^. p27, which is otherwise an intrinsically disordered protein (IDP)^14^,^15^,^16^, exhibits an extended conformation in this ternary complex. The crystallized kinase inhibitory domain of p27 contained 69 amino acids and is comprised of a rigid coil (residues 26–35), an amphipathic α-helix (residues 38–59), an amphipathic β-hairpin (residues 61–71), a β-strand (residues 75–81), and a 3_10_ helix (residues 85–90). The rigid coil with LFG motif (the conserved sequence of Leu-Phe-Gly) binds to a shallow groove nested by the α1, α3, and α4 helices of the cyclin-box repeat of CyclinA through the hydrogen-bonding interactions. The amphipathic α-helix also packs against the hydrophobic surface of the α4 and α5 helices of the cyclin box, but relatively less tightly. The amphipathic β-hairpin, β-strand, and the 3_10_ helix bind to CDK2. The β-hairpin packs against the N-terminal β-sheet of CDK2 in a β-sandwich arrangement. The β-strand incorporates into the CDK2 N-terminal β-sheet and produces a p27-CDK2 hybrid β-sheet. Following this, the 3_10_ helix inserts deep into the CDK2 catalytic cleft and completely blocks the access of ATP. Thus, the crystal structure of p27/CDK2/CyclinA does not contain the ATP molecule, nor it contains the glycine rich loop, the so-called G loop, that aligns the substrate and ATP correctly for a smooth transfer of the γ-phosphate^11^,^17^,^18^,^19^.

The mechanism of CDK2 reactivation is proposed to involve the phosphorylation of p27 tyrosine residues by the tyrosine kinases. p27 contains three tyrosine residues – Y74, Y88, Y89 in the CDK inhibitory domain. Out of these, Y88 and Y74 are shown to have higher propensity for phosphorylation^13^. While ABL and LYN kinases predominantly phosphorylate Y88, SRC kinase can phosphorylate both Y88 and Y74^12^,^13^. NMR data suggest that Y88 phosphorylation can eject the 3_10_ helix of p27 from the CDK2 catalytic pocket, thus making CDK2 partially active^12^. Interestingly, this structural change transforms p27 from a CDK inhibitor to a CDK substrate, whose T187 residue in C-terminal gets phosphorylated by the same CDK/Cyclin complex^8^. The role of Y74 phosphorylation on CDK2 activity is, however, not yet very clear. While some studies have shown that on an average only one tyrosine per inhibitor molecule becomes phosphorylated, other reports speculated that phosphorylation of p74 together with Y88 may reverse CDK2 fully active by promoting the dissociation of p27 from the ternary complex^13^.

In this work, we attempt to explore the detailed mechanism of CDK2 reactivation by Y88 phosphorylation through classical and accelerated molecular dynamics (MD) simulation techniques. These methods allowed us to elucidate the structural and dynamical changes in CDK2 upon p27 phosphorylation at atomic level. Results suggest that Y88 phosphorylation (pY88) ejects the 3_10_ helix from the CDK2 catalytic cleft, without much disruption of other interactions of p27 with CDK/Cyclin. Upon this ejection, the catalytic cleft of CDK2 becomes very similar to that of the active pCDK2/CyclinA complex and completely accessible to ATP. Molecular docking and free energy calculations show that ATP could bind to the phospho-p27/CDK2/CyclinA complex as efficiently as it binds to the active pCDK2/CyclinA binary complex. More interestingly, the CDK2 G-loop, which could not be traced in the p27/CDK2/CyclinA crystal structure due to poor electron density, appears in correct position during MD simulations for proper alignment of ATP. The transition of the p27-bound inactive to p27-unbound active CDK2/CyclinA complex appears to follow a two-step mechanism, where the breaking of the p27-CDK2 hybrid β-sheet in the first step is followed by the rate-limiting step of p27 3_10_ helix ejection from the CDK2 catalytic pocket.

## Results and Discussion

To start with, we performed classical MD simulations (cMD) of unphosphorylated and Y88-phosphorylated p27 bound CDK2/CyclinA complexes. The simulation of the unphosphorylated complex was initiated from the crystal structure of p27/CDK2/CyclinA (PDB ID: 1JSU)^11^. The crystal structure had twelve missing residues from the N-terminal of CDK2 that constitute the G-loop and β1 strand in the active CDK2/CyclinA complex. Due to the functional significance of the G-loop, the missing residues were incorporated into this crystal structure. The modified structure was equilibrated in explicit water *via* a 25 ns MD run. This equilibrated structure was then used to generate the pY88-p27/CDK2/CyclinA complex by adding a phosphate group to the Y88 residue of p27. This latter structure was also subjected to initial relaxation followed by a MD run of 25 ns under NPT conditions for equilibration. Subsequently, both the unphosphorylated and phosphorylated systems were further simulated for 200 ns each to generate data for analysis (Table S1).

### Binding of p27 to CDK2/CyclinA is very tight

Before performing analyses on the simulation trajectories, we wanted to make sure that the systems were well equilibrated. Hence, we monitored the root mean square deviations (RMSD) of both the complexes from the crystal conformation and the results are shown in Fig. S1. As the figure shows, the crystal structure derived conformation of p27/CDK2/CyclinA attained stability very quickly, within the initial 5 ns. However, the pY88-p27/CDK2/CyclinA system required nearly about 60 ns to stabilize due to the added phosphate group. Hence, the subsequent analyses were performed on the final 140 ns data of both the systems.

Comparison of the time-averaged conformations with the crystal structure revealed that except for the phosphorylated Y88 (pY88) in the pY88-p27/CDK2/CyclinA system, all other p27 residues maintain their native contacts with CDK2/CyclinA in both complexes (Fig. S2a,b). Computed total interaction energy between p27 and CDK2 in the simulated structures, therefore, showed only a minute difference from the crystal conformation value of −144.08 kcal/mol (Fig. S2c and more details below). However, the phosphorylated Y88 and the neighbouring residues in p27: 3_10_ helix exhibited significantly larger fluctuations compared to the unphosphorylated complex (Fig. S3). These observations, therefore, suggest that the binding of p27 to CDK2 is tight (Fig. S2), even though the Y88 phosphorylation tends to weaken some of the interactions (Fig. S3). However, no observed flip-out of the 3_10_ helix, as reported in NMR experiments^12^, implies that the transition could be slow.

A closer look revealed that Y88 becomes solvent exposed upon phosphorylation, as shown in Fig. S2b, mainly due to the repulsive interactions of its attached phosphate group with CDK2 active site residue, E81. Although this induces certain loss of secondary structures in p27, its native interactions with CDK2, including those from the 3_10_ helix region remain intact (Fig. S2c). For example, residue P85 from p27 maintained van der Waals contacts with L134 of CDK2 and p27: E86 maintained hydrogen bonding interactions with CDK2: Q131 residue. Similarly, the hydrogen bond between p27: R93 terminal amine and CDK2: Asp 127 side chain carboxyl group was intact. Interestingly, new hydrogen bonds were found to be formed between p27: S83 and CDK2: D86 and also between p27:K81 and CDK2: G16 backbone carbonyl, as a result of the phospho-Y88 flip (not shown). Thus, even though Y88 is dislocated upon phosphorylation, other p27 residues maintained their interactions with CDK2. It is possible that these interactions between the p27 and CDK2 interfacial residues constitute a low-energy conformation, which is separated from the NMR reported flipped out conformation of 3_10_ helix by high energy transition states. Recent studies have indeed shown that kinetically distinct conformations of proteins are separated by large energy barriers^20^,^21^.

Such a transition with large energy barrier is unlikely to be captured by the time scale amenable to classical MD simulation method^22^,^23^. Hence, we adopted an advanced molecular dynamics technique, called accelerated molecular dynamics (aMD) that accelerates the transitions by modifying the potential energy surface by adding a bias potential to the true potential such that the escape rates from the energy wells are enhanced^24^,^25^. Two biggest advantages of this method over the other enhanced sampling techniques are that it does not require a prior knowledge of the nature of the free energy landscape, nor it requires the specific prior definition of a reaction coordinate^24^. Hence, last decade has seen an increasing application of this technique to explain multitude of biological phenomena that occur in the micro- to millisecond time scale^22^,^23^,^26^,^27^. Here, we employed this technique to the ternary p27/CDK2/CyclinA complexes with the hope of overcoming the energy barriers for transitions and subsequently to capture the active conformation of CDK2. If successful, the study will pave way for understanding the detailed mechanism of CDK2 reactivation by p27 phosphorylation.

### Unbinding of phosphorylated-p27 from CDK2/CyclinA requires barrier crossing

Accordingly, we have performed aMD simulations on both the unphosphorylated (as the control) and phosphorylated p27/CDK2/CyclinA complexes (Table S1). The aMD simulations were started from the final structures of the 200 ns cMD simulations as discussed above. With the progression of the aMD simulations, the protein components were seen to exhibit significantly larger conformational variations than the cMD simulations. Figure 2 presents the RMSD distribution of the entire set of conformations that the proteins have explored during the long cMD and aMD simulations. Clearly, aMD simulations have sampled a wider range of protein conformations. More importantly, aMD simulation of pY88-p27/CDK2/CyclinA system shows a broad range of conformations of p27 whose RMSD spans up to 12 Å (Fig. 2d, red plot). Representative conformations of p27, from the two distinct ensemble of structures in Fig. 2d, are shown in Fig. S4 and discussed below. However, the range of distributions of CDK/Cyclin conformations was very similar to the p27-phosphorylated classical MD simulations, suggesting that the observed larger dynamics in p27 may not be an artefact of the enhanced sampling method adopted here. It is also worth noticing here that the Y88 phosphorylation itself (i.e. without accelerating the simulation by boost potential) could increase the conformational sampling of the proteins, as evident from the comparison of probability distributions in Fig. 2a,b. The RMSD distribution in this case, *i.e*. phosphorylated cMD, is very similar to that of the control aMD simulation (compare Fig. 2b,c). Even the distribution of CDK/Cyclin in this simulation (i.e. the phosphorylated cMD) matches well with that in the phosphorylated aMD simulation (compare Fig. 2b,d). These results from cMD *versus* aMD, thus, suggest that p27 unbinding from CDK2/CyclinA might require a barrier crossing and Y88 phosphorylation plays the primary role in the process of 3_10_ helix ejection from CDK2 catalytic cleft.

To visualize the conformational changes more explicitly, we have presented the three dimensional (3D) structures of the protein complexes from aMD simulations in Fig. 3. As evident, the ternary complex in unphosphorylated state retains the native conformation, with the 3_10_ helix and other p27 domains maintaining the interactions with CDK2 catalytic cleft (Fig. 3a). Even the secondary structural elements of p27 were intact at such a high boost potential, suggesting that the applied potential is conducive. Conversely, the structure of the phosphorylated complex from aMD simulation exhibits significantly altered arrangement of the constituent proteins (Fig. 3b). Majority of the p27 interactions with CDK2 were lost, even though the CDK2-CyclinA interfacial contacts were maintained (shown later). The packing of p27 β-strand with CDK2 β-sheet became weaker and the 3_10_ helix completely ejected out of the CDK2 catalytic cleft (Fig. S5). The time evolution of this exit process is shown in Fig. S5, where it is clear that only the 3_10_ helix and β-strand regions of p27 undergo significant changes. Remaining motifs of the structure show considerable overlap with the starting crystal conformation and the simulation-generated intermittent conformations (Fig S5b). Also, the ejection of the 3_10_ helix was not an instant process. As Fig S5a shows, there was a considerable delay (~80 ns of aMD simulation time) in the occurrence of the unbound state of p27 from the bound p27-CDK2/CyclinA complex. This might imply crossing of a high energy barrier for this structural transition and will be discussed at length in the next section. However, once formed, the unbound p27 maintains a ‘flip out’ conformation of its 3_10_ helix for the rest of the simulation time. This ‘flip out’ conformation of p27 at steady state matches very well with the NMR chemical shift data that suggested the ejection of p27 3_10_ helix from the ATP binding pocket of CDK2^12^.

### p27 unbinding does not affect CDK2/CyclinA contacts

As a consequence of these structural changes, the interfacial contacts between p27 and CDK2 reduced significantly as shown in Fig. 4a. However, this reduction was mostly confined around the 3_10_ helix region and the β-strand region of p27. The β-hairpin and domain LH of p27 maintained similar contacts with CDK2 as in the unphosphorylated complex. Also, the contacts of p27 with Cyclin residues remain very similar (Fig. S6). Computed buried surface area in the unphosphorylated and phosphorylated p27/CDK2/CyclinA complex was found to be 5737 ± 65 Å^2^ and 4665 ± 112 Å^2^, respectively (reported buried area in the crystal structure of the unphosphorylated complex was 5752 Å^2^)^11^. Thus, there was a loss of 1072 ± 82 Å^2^ (18.7%) of buried area upon phosphorylation, out of which the 3_10_ helix alone accounted for the loss of 637 ± 56 Å^2^ (11.1%) of the area. This corroborates very well with the experimental finding that local disruption due to the ejection of 3_10_ helix does not affect the binding of other p27 domains to CDK/Cyclin. It is also worth noticing here that the p27 β-strand and β-hairpin regions show a new set of contacts with CDK2 in the phosphorylated complex (red box in Fig. 4). A closer look revealed that this was due to the spontaneous appearance of CDK2 β1 strand and G-loop near the ATP binding pocket, which were missing in the p27/CDK2/CyclinA crystal structure due to their poor electron density. This will be discussed in detail in the next section. More interestingly, the contacts between CDK2-CyclinA were found to remain unperturbed in this phosphorylated complex and strongly resemble that in the crystal structure of the active CDK2/CyclinA complex (PDB ID:1JST)^2^. This is shown in Fig. 4b. It is to be recalled that the catalytic efficiency of CDKs primarily depends on their interactions with the activators, the Cyclins.

The loss of p27-CDK2 interfacial contacts can be explained from the analysis of residue-level interactions. Figure 5 highlights some of the most significant structural changes that the p27 residues experienced due to Tyr88 phosphorylation. In unphosphorylated state, the aromatic ring of p27:Tyr88 makes van der Waals contacts with CDK2:Phe80, Phe82 and its hydroxyl group involves in H-bonding interactions with the backbone carbonyl of CDK2:Glu81 and backbone amide of CDK2:Leu83 (Fig. 5a). Upon phosphorylation, phosphorylated Tyr88 (pY88) loses these H-bonding interactions and becomes solvent exposed, primarily due to the repulsive interactions of its phosphate group with the negatively charged side chain of CDK2:Glu81. However, this change does not affect the interaction of other residues of p27: 3_10_ helix with CDK2 pocket residues and, hence, the two proteins continue to remain in the bound form. A closer look into the system’s evolution (Movie S1) exhibits that the subsequent breaking of p27-CDK2 interactions was initiated by the loss of p27-CDK2 hybrid β-sheet contacts, where the β-strand of p27 was gradually displaced out of the hybrid β-sheet. The backbone-backbone hydrogen bonds between p27: V79 and CDK2: V18, p27: E78 and CDK2: Y19, p27: Q77 and CDK2: K20 etc., that were present in the crystal structure (Fig. 5b), were lost in the phosphorylated complex (Fig. 5c). The hydrophobic packing between p27: V79, L84 and CDK2: V18 side chains, which was found in the crystal structure, was also lost in the phosphorylated complex (Fig. 5b,c). The hydrogen bonds between p27: S83 and CDK2: D86 and between p27: K81 and CDK2: G16, those were formed during phospho-Y88 flip, were also lost. These loss-of-contacts unpack the p27 β-strand from p27-CDK2 hybrid β-sheet and trigger the exit of 3_10_ helix from the CDK2 catalytic pocket. Consequently, the major interactions that maintain the 3_10_ helix in the CDK2 pocket, such as the van der Waals contacts between p27: P85 and CDK2: L134, H-bond interactions between p27: E86 and CDK2: Q131, and between p27: R90 carbonyl and CDK2: K33 side chain were weakened and 3_10_ helix ejects out of the CDK2 pocket (Fig. 5d).

In a nutshell, the phosphorylation at Y88 flips out pY88 that induces a cascade of changes, wherein first p27 β-strand unpacks from the p27-CDK2 hybrid β-sheet, which in turn triggers the exit of 3_10_ helix from the CDK2 catalytic pocket. These observations are consistent with Fig. S5, which indicated a time lag of about 80 ns before the 3_10_ helix ejects out of the pocket. Computed energetics by Molecular Mechanics Generalized Born Surface Area (MMGBSA) method also shown that the binding of p27 β-strand with CDK2 β2 strand in the hybrid β-sheet is weaker than the binding of 3_10_ helix with the CDK2 catalytic pocket (−50.42 ± 0.05 kcal/mol *versus* −71.91 ± 0.04 kcal/mol out of the total p27-CDK2 interaction energy of −144.08 kcal/mol), and thus the breaking of hybrid β-sheet is presumably the first step to initiate the p27-CDK2 dissociation process. Interestingly, this process of breaking of the hybrid β-sheet was noted to be highly activated by the added sequence of twelve residues that constitute the G-loop and β1 strand in active CDK2.

To strengthen our finding that the breaking of hybrid p27/CDK2 β-sheet constitutes the first step of p27 unbinding, we have performed site-specific alanine mutational study, wherein we have mutated each of the p27 β-strand residues that were found to play a key role in p27 unbinding and subsequently simulated them individually. A minimum of 80ns aMD simulation was performed on each of the following mutations: Q77A, E78A, K81A, S83A, L84A, P85A. Among the mutated residues, K81A induced a faster unbinding of p27 from CDK2/CyclinA complex during the simulation of the phosphorylated complex. The breaking of hybrid β-sheet and 3_10_ helix took place in 45 ns compared to 80 ns in WT (Fig. S7). Among others, S83A and P85A mutations also have indicated the unbinding of p27 from CDK2/CyclinA complex, but to a lesser extent.

### Spontaneously formed CDK2 G-loop aligns ATP correctly

**Figure 1:**
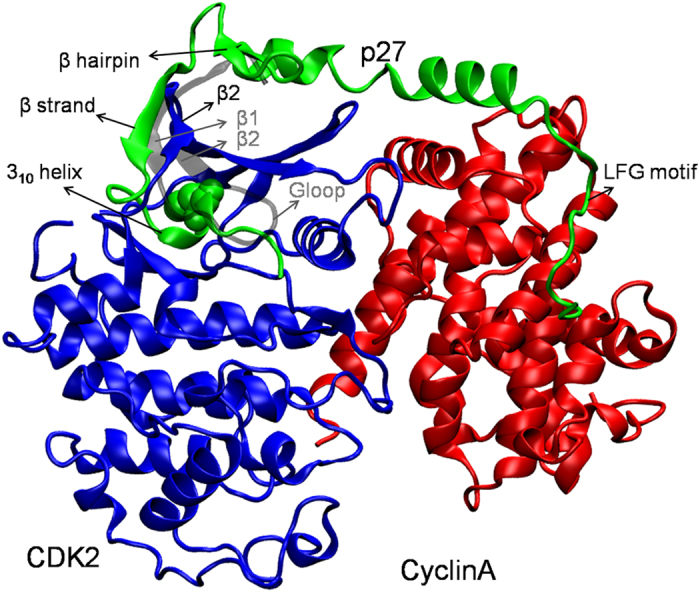
Crystal structure of p27/CDK2/CyclinA complex (PDB ID: 1JSU^11^). Color scheme is, CDK2 blue, Cyclin A red, and p27 green. Important regions of p27 are labeled and the location of Tyr88 in 3_10_ helix of p27 is shown in sphere representation. The position of β2 strand of CDK2, which forms hybrid β-sheet with p27 β-strand is shown and labeled. Notably, the β1 strand and G-loop of CDK2 were missing in this structure. For comparison, the position of β2 strand and that of the G-loop and β1 strand in active CDK2 is shown (in grey, also labelled in grey) from the crystal structure of CDK2/CyclinA complex (PDB ID: 1QMZ^28^) by superposing the two crystal structures.

**Figure 2:**
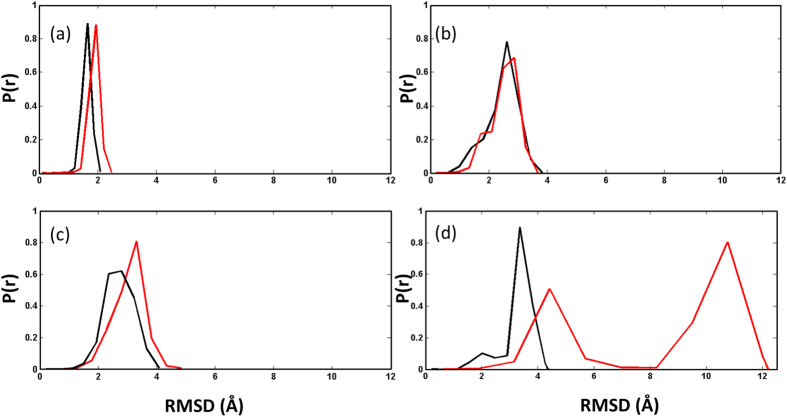
Conformational variations in p27. RMSD distributions of p27 (red) and CDK2/CyclinA (black) are shown for (**a**) cMD unphosphorylated, (**b**) cMD phosphorylated, (**c**) aMD unphosphorylated and (**d**) aMD phosphorylated systems. RMSDs are calculated with respect to the C_α_ atoms of the protein residues.

**Figure 3:**
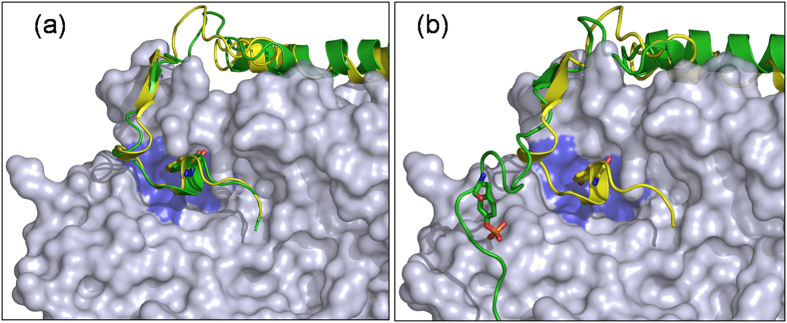
Structure of p27 upon phosphorylation. Time-averaged conformations of p27 (green) and CDK2/CyclinA (ice blue) are shown for (**a**) p27/CDK2/CyclinA and (**b**) pY88-p27/CDK2/CyclinA complexes from aMD simulations. For comparison, the p27 conformations from aMD are superposed on the crystal conformation (yellow). Phosphorylated Y88 in p27 is shown in sticks, with P in orange and O in reds. CDK2 catalytic pocket is highlighted in blue.

**Figure 4:**
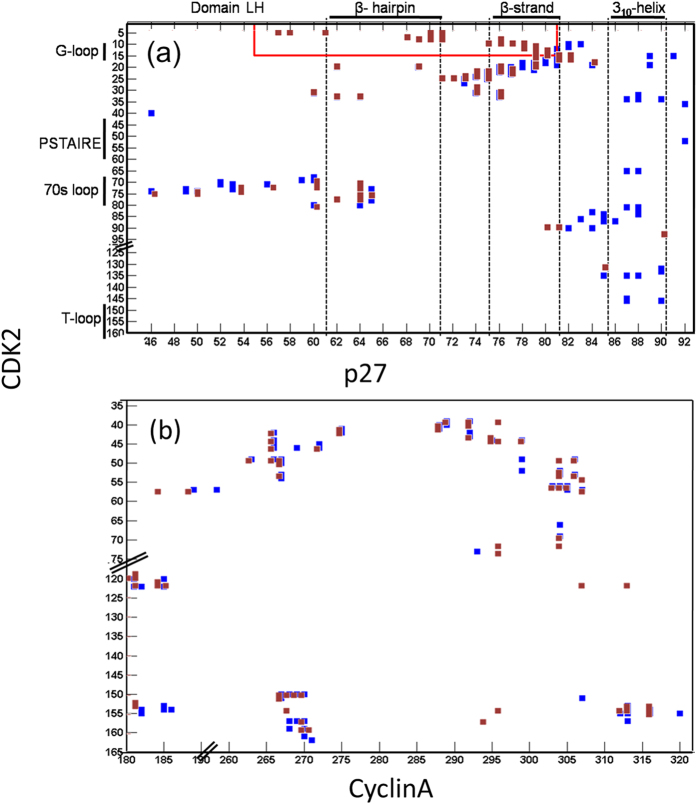
Modulated p27/CDK2 interfacial contacts upon phosphorylation. (**a**) Comparison of p27/CDK2 interfacial contacts in the p27 unphosphorylated (blue) and p27 phosphorylated (brown) complex from aMD simulation results. (**b**) Comparison of CDK2/CyclinA interfacial contacts in the functionally active form of CDK2/CyclinA (blue, from crystal structure: 1JST^2^) and phosphorylated-p27/CDK2/CyclinA complex (brown, from this aMD simulation). Each square represents contact between a pair of residues exceeding a contact area 5 Å^2^. The red box in (**a**) highlights the region of new contacts that are generated due to folding of CDK2 G-loop and β1-strand.

To be recalled, the sequence of G-loop and β1 strand was missing in the p27-bound crystal structure of inhibited CDK2/CyclinA complex (Fig. 1). In the phosphorylated complex (from our aMD simulation), the added sequence was found to dislocate p27 β-strand from the hybrid β-sheet and attempted to generate the N-terminal β-sheet and G-loop that the active CDK2 possesses^2^. As Fig. 6a shows, the p27 β-strand is dislodged (the strand in green) and the β2 strand of CDK2 (the strand in magenta), which was 8.5 Å away in the p27/CDK2/CyclinA crystal structure, takes up a position that is similar to that in the active CDK2/CyclinA complex. The RMSD of this reorganized strand from the structure of the active CDK2/CyclinA complex is now less than 1.3 Å. Computed distance values of the major interacting residues of β2 with the neighbouring β3 strand residues also show the reproduction of CDK2 N-terminal β-sheet, where the initial distances of β2- β3 residues V18CA-K33CA, Y19N-L32O and V17O-K34N reduced from 7.35 Å, 4.52 Å and 8.22 Å to 4.85 Å, 2.82 Å and 3.22 Å, respectively, which mimic the crystal structure distance values of 4.69 Å, 3.08 Å and 3.19 Å in the active CDK2/CyclinA complex^2^.

Even more importantly, the G-loop with characteristic U conformation that caps the ATP/substrate binding pocket in active CDK2, appeared spontaneously in place upon p27 dislocation. The cross-links between the residues in this loop were found to be similar to that in the crystal structure of active CDK2. For example, the important loop-forming inter-residue distances of G11CA-V18CA, E12CA-G17CA, G13CA-G16CA and T14CA-Y15CA matched reasonably well with the crystal structure values. The respective distances were 3.79 Å, 6.64 Å, 5.95 Å and 6.29 Å *versus* 3.85Å, 5.51 Å, 3.84 Å and 5.19 Å in crystal^2^. Although the secondary structure of β1-strand could not be produced in the stipulated time of the simulations, the distances of β1-β2 strand residues V8 and K21, Q9 and Y20, K10 and V19 were found to reproduce well the qualitative trend of the crystal structure values (9.9 Å, 6.09 Å and 6.96Å *versus* 7.21 Å, 6.02 Å and 5.05 Å in the crystal structure of active CDK2)^2^. Thus, it is apparent that the dislocated β2 strand and missing G-loop and β1 strand in the inhibited p27/CDK2/CyclinA complex could be reformed to active-like conformation by p27Y88 phosphorylation (magenta loop in Fig. 6a). However, a similar conformational change could not be observed in the control simulations (systems 1–3 in Table S1), where the added sequence of residues continued to remain solvent exposed as a random coil and never could engage in interactions with core CDK2 residues (similar to orange loop in Fig. 6a).

To verify further if these rearrangements indeed produced the active conformation of CDK2, which takes up ATP in the catalytic cleft for the transfer of phosphate to the substrate, we performed docking studies of ATP into our aMD-generated phospho-p27/CDK2/CyclinA complex. Noting the presence of divalent ions and crystal water in the ATP binding pocket of active CDK2/CyclinA complex, we have included Mg^2+^ with crystal water in the obtained structure by superposing it with the available crystal structure of the active complex (PDB ID: 1QMZ^28^), prior the docking trials. Protein-ligand docking was performed using AutoDock 4.2.6 with the consideration of ligand flexibility. To introduce the receptor flexibility, we have adopted the principle of Relaxed Complex Scheme proposed by McCammon and coworkers^29^, according to which the flexible ATP was docked into a series of CDK2 conformations obtained from the aforementioned simulations of phospho-p27/CDK2/CyclinA and native p27/CDK2/CyclinA complexes. The average binding energy of ATP over such 25 CDK2 conformations (generated at 1 ns interval from the final 25 ns aMD data) is shown in Table 1. As the table indicates, the phospho-p27/CDK2/CyclinA complex indeed becomes active with the free energy of ATP binding is very similar to that of the active CDK2/CyclinA binary complex^30^. On the contrary, the binding of ATP to the p27-bound inhibited complex is very unfavourable. Fig. S8 presents a close-up view of the time-averaged orientation of ATP in the CDK2 catalytic pocket. It is evident from this figure that the known pose of ATP in the crystal structure of active CDK2 is very well recovered in the phospho-p27 complex (compare Fig. S8a,b). Moreover, the interactions between ATP and CDK2 active site residues that maintain ATP in the reactive state, e.g. H-bonds between N1, N6 nitrogens of ATP and CDK2: L83N, E81O sites, van der Waal’s interactions of ATP purine ring with CDK2: Leu134, electrostatic interaction of ATP phosphates with CDK2: Lys33 side chain, and associated coordination of Mg^++^ with ATP phosphate oxygens, CDK2: Asn132, Asp145 side chains and with bound water could also be reproduced in the phospho-p27/CDK2/CyclinA complex^2^,^17^,^18^. Such a close resemblance in orientation and contacts manifests in very similar binding of ATP in the phospho-p27/CDK2/CyclinA and active CDK2/CyclinA complexes (Table 1). Thus, a detailed mechanism of CDK2/CyclinA reactivation upon phosphorylation of the regulatory protein, p27 is established. However, an effective blocking of the CDK2 catalytic cleft by p27 3_10_ helix makes ATP binding to p27/CDK2/CyclinA very unfavourable (Fig. S8c).

### Displacement of p27 3_10_ helix is the rate-limiting step

**Figure 5:**
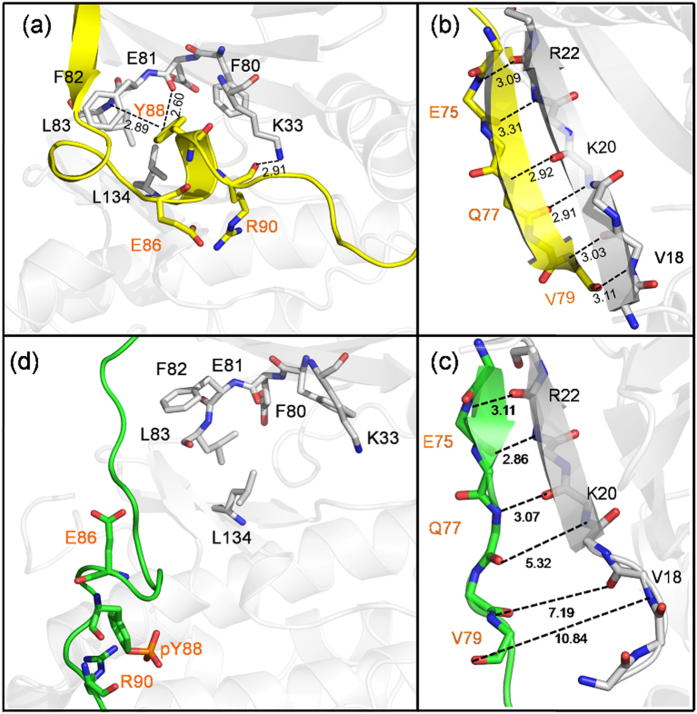
Structural changes in unphosphorylated and phosphorylated complexes at the p27/CDK2 interface. Unphosphorylated complex shows large number of hydrogen-bonded interactions (shown by dotted lines) (**a**) between 3_10_ helix of p27 and CDK2 residues in the CDK2 catalytic cleft and (**b**) between β-strand of p27 and β2-strand of CDK2 in the hybrid beta-sheet. The majority of these interactions were lost upon phosphorylation, as shown in (**c,d**). Unphosphorylated and phosphorylated p27 are shown in yellow and green color, respectively. The CDK residues have been labeled in black and p27 residues are in orange. The numbers indicate the H-bond distances.

**Figure 6:**
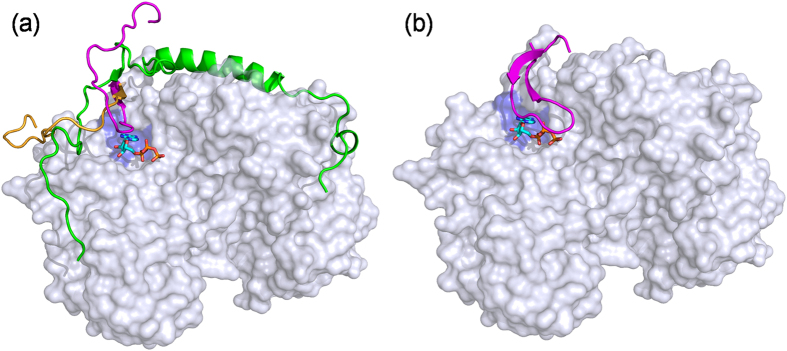
Comparison of ATP binding in simulated complex and crystal structure. Comparison of the structures of (**a**) ATP-docked simulated complex of pY88-p27/CDK2/CyclinA from our aMD simulations and (**b**) ATP-bound active CDK2/CyclinA crystal structure complex (PDB ID: 1JST). In (**a**), the relocation of crystal-structure-missing CDK2 β1-strand and G-loop region to its functional position is also shown, with the initial and final position in orange and magenta colour, respectively. The dislodged p27 due to this relocation of G-loop is shown in green. Other colour scheme includes: CDK2 in white, cyclin A in cyan, CDK2 ATP binding pocket in ice blue, and ATP in sticks.

**Figure 7:**
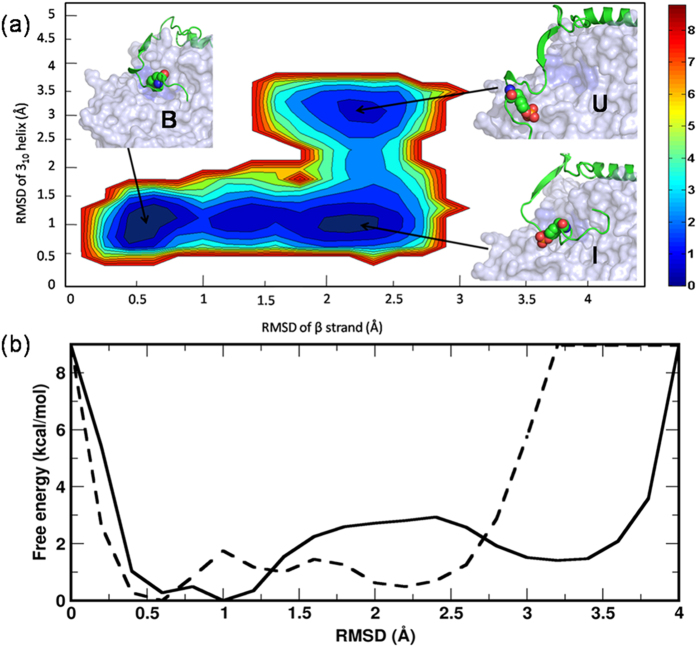
Free energy profile of the unbinding of phosphorylated p27. (**a**) The unfolding free energy landscape of pY88-p27 from CDK2/CyclinA complex with associated color scale. Representative structures of the three major states are shown - the bound state (state-B), intermediate state (state-I), and unfolded state (state-U). (**b**) The 1D free-energy profiles of p27 unbinding based on C_α_-RMSD of its β-strand (dashed lines) and 3_10_ helix (solid line) as obtained from Boltzmann reweighting distribution.

Lastly, we were tempted to delineate the free energy landscape and estimate the energy barriers of transition of the p27 bound CDK2/CyclinA inhibited complex to the p27 unbound CDK2/CyclinA active complex. Two-dimensional profile of the free energy landscape is constructed by projecting the population of states onto the plane defined by the C_α_-RMSDs of the β-strand and 3_10_ helix of p27. As our aforementioned discussion suggests (Fig. 5, Movie S1), the evolution of p27 β-strand and 3_10_ helix could describe the entire process of p27 unbinding from CDK2, and hence chosen as the reaction coordinates to draw the free energy profile. Figure 7 shows the Boltzmann reweighted distribution of states, where each frame of the aMD trajectory was Boltzmann reweighted by its respective boost factor to recover the correct canonical ensemble. As the free energy landscape in Fig. 7a shows, there exists three distinct regions that correspond to (i) the p27-CDK2/CyclinA bound state, state-B (R_β_ < 0.7 Å and R_310_ < 1.3  Å), (ii) the intermediate state where the p27-CDK2 hybrid β-sheet was broken, state-I (R_β_ > 1.7 Å and R_310_ < 1.3 Å) and (iii) the unbound state where the p273_10_ helix completely ejected out of the CDK2 catalytic pocket, state-U (R_β_ > 1.7 Å and R_310_ > 2.8 Å). The lowest energy state corresponding to the p27-bound CDK2/CyclinA crystal conformation at R_β_ = 0.5 Å and R_310_ = 1.0 Å was considered as the reference point (ground state), with free-energy value 0.0 kcal/mol.

As the free energy profile indicates, the state-B reaches to the state-U by crossing two energy barriers. First, it crosses a minor barrier of about 1.74 kcal/mol to reach a metastable state (state-I) by unpacking the hybrid p27-CDK2 β-sheet (Fig. 7b dashed line). As this figure indicates, the metastable state has an energy 0.5 kcal/mol higher than the ground state. Subsequently, the system from this state crosses a major barrier of 2.94 kcal/mol to reach to state-U by displacing the p27 3_10_ helix out of the CDK2 catalytic pocket (Fig. 7b solid line), amounting a total barrier height of 3.44 kcal/mol for p27 unbinding. Thus, the free energy profile is suggestive of a two-step mechanism for p27 unbinding, where the displacement of 3_10_ helix stands as the rate-limiting step. This result is also consistent with the stepwise folding/unfolding mechanism of various other proteins from different family, such as titin, villin headpiece subdomain etc^31^,^32^,^33^. For comparison, we have also delineated the free energy landscape of the unphosphorylated p27/CDK2/CyclinA complex from aMD simulation data (control system) and the results are shown in Fig. S9. The free energy landscape exhibited a narrow range of conformations (R_β_ < 0.7 Å and R_310_ < 1.3  Å) that correspond to the p27-CDK2 bound state and no p27-unbinding from CDK2 surface could take place.

It has been shown very recently that reweighting of aMD simulations using Maclaurin series expansion can improve the free energy profiles by suppressing the energetic noise due to Boltzmann reweighting factors in exponential average^34^. Hence, we also reweighted the aMD simulation data by using Maclaurin series expansion (cumulant expansion on the first order) and plotted the free energy profile. Results from both algorithms were found to bear high resemblance (Fig. S10). However in accordance with the earlier reports, even though the Maclaurin series expansion suppressed the energetic noise, it produced energy barriers that are lower than the calculated values from exponential average (1.07 and 2.56 kcal/mol in Maclaurin expansion *versus* 1.74 and 2.94 in exponential average). The energy minima were also found to shift a little from the positions in Boltzmann reweighted distribution. Nevertheless, both methods converge to predict a two-step unbinding mechanism of p27 from CDK2/CyclinA complex.

## Discussion

The activity of cell-cycle protein, CDK2 is closely regulated by the binding and unbinding of KIP/CIP family protein, p27. Recent studies have shown that the binding of p27 to CDK2 inhibits CDK2 activity, while phosphorylation of certain p27 tyrosine residues resumes the activity of CDK2. Even though NMR chemical shift data indicated the ejection of p27 3_10_ helix from CDK2 catalytic site, the detailed mechanism of p27 unbinding from the inactive p27/CDK2/CyclinA ternary complex and subsequent mechanism of CDK2 reactivation are unknown. Our all-atom classical and accelerated MD simulation data show that p27 unbinding from CDK2 follows a two-step mechanism, where p27:Y88 phosphorylation first disrupts the p27/CDK2 hybrid β-sheet and subsequently ejects the p27 3_10_ helix from CDK2 catalytic cleft. The barrier height of the unbinding process was ~3.5 kcal/mol and ejection of 3_10_ helix constituted the rate-limiting step. Upon p27 unbinding, CDK2 regained significant catalytic activity as exemplified by its retention of specific interfacial contacts with CyclinA present in the active CDK2/CyclinA complex. More notably, the missing β1-strand and G-loop in inhibited CDK2 crystal structure were reproduced in place to allow proper ATP binding in pY88-p27 state. The calculated value of free energy of ATP binding to pY88-p27/CDK2/CyclinA was very similar to the experimental value for the active CDK2/CyclinA complex. Thus, a detailed mechanism of p27 unbinding for CDK2 reactivation became evident from this study.

## Methods

To start with, we performed classical MD simulations (cMD) of unphosphorylated and Y88-phosphorylated p27 bound CDK2/CyclinA complexes. The simulation of the unphosphorylated complex was initiated from the crystal structure of p27/CDK2/CyclinA complex (PDB ID: 1JSU)^11^. In this structure, the missing twelve residues (sequence MENFQKVEKIGE) at the CDK2 N-terminal were modeled using the InsightII graphics package^35^. The hydrogens for heavy atoms in the protein residues were added by leap module in Amber 12.0 package^36^. Subsequently, an energy minimization for 1000 steps using the steepest descent and another 1000 steps using the conjugate gradient algorithm was carried out. The protonation states of histidines - HID or HIE - were determined by the local hydrogen bonding network using WHATIF^37^. After initial relaxation of the added atoms in gas phase, the structures were solvated in a cubic periodic box of explicit water with water molecules extending 10 Å outside the protein on all sides. The 3-site TIP3P model was chosen to describe the water molecules^38^. Na^+^ ions were added to neutralize the system. Aqvist parameters were chosen for sodium^39^. In the primary simulation of phosphorylated complex, the total number of atoms was 82293.

To remediate any overlap or close proximity of the added sequence of N-terminal missing residues and added water and ions with the p27/CDK2/CyclinA crystal conformation, an extensive set of minimization and thermalization was performed. For this, a further 1000 steps of conjugate gradient minimization was performed followed by successive heating to 310K with an temperature increment of 25K and maintaining Cα restraints for a total duration of 5 ns. The resulting structure was further minimized after removing the restraints and heated to 310K in 10 steps of 1 ns each. Then the system was equilibrated for 25 ns in NPT ensemble with a simulation time step of 2 fs. During this period, the energy components and RMSD of the structures relative to the crystal conformation were seen to converge. The simulation was extended further for another 200 ns and designated as the control system-1. As the second control, Tyr88 in p27 of the above-mentioned equilibrated structure was phosphorylated by leap programme in AMBER using the contributed dataset in amber^40^. This structure was also subjected to initial relaxation followed by a MD run of 25 ns under NPT conditions for equilibration. Subsequently, it was also simulated for 200 ns to generate data for analysis. Table S1 lists all the systems simulated here. SHAKE was used to constrain bond lengths between heavy atoms and hydrogens. The long-range electrostatic interactions were treated by using Particle-Mesh Ewald sum and SHAKE was used to constrain all bonds involving hydrogen atoms^41^,^42^. Amber12 molecular dynamics simulation package with Amber ff99SB force field was used^43^. Calculation of free energy for ATP binding was done by using Autodock 4.2.6^44^.

### Accelerated molecular dynamics

We have also performed accelerated MD simulations on both p27/CDK2/CyclinA and its phosphorylated analogue as listed in Table S1. Accelerated MD (aMD) is an advanced MD simulation technique that can capture high and low energy transition states by modifying the potential energy surface through the addition of a non-negative boost potential ∆V(r) to the original potential V(r), whenever V(r) is below a pre-defined energy level E^24^,^25^:


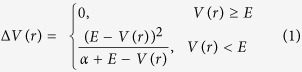


where, α modulates the depth and the local roughness of the energy basins on the modified potential. In order to enhance the sampling of internal and diffusive degrees of freedom concurrently, a dual boosting approach was employed by applying one boost potential to the torsional terms and another to the total potential as follows:


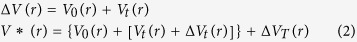


where, V_t_(r) is the potential of the torsional terms. ∆V_t_(r) and ∆V_T_(r) are the boost potentials applied to the torsional terms and the total potential energy, respectively, and both follow the same definition as in Eq. (1). The boost parameters were estimated from the average dihedral and potential energy of the systems, as obtained by running short classical molecular dynamics (cMD) simulations^30^,^32^. Following the implementations in AMBER12, the torsional boost parameter was calculated as a sum of average dihedral energy value (as obtained from MD) +3.5 times the number of residues. The alpha parameter in eq. 1 is then set to 0.15 times the contribution of the latter term. For the total energy, alpha was set to be 0.15 times the total number of atoms and the boost energy was set to be the sum of average total potential energy (as obtained from cMD) + alpha. The corrected canonical ensemble average of the system is then obtained by simply reweighting each point in the configuration space by exp[β(∆V_t_(r) + ∆V_T_(r))] for the Boltzmann reweighting^22^. We have also used Maclaurin series expansion to the 10^*th*^ order for reweighting the aMD simulations^34^. For Maclaurin series expansion, the boost potential with the reweighting factor can be written as: 
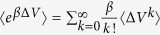


### Binding energetics

Binding energetics between p27 and CDK2 was calculated as free energy of their binding using Molecular Mechanics Generalized Born Surface Area (MMGBSA) approach^45^. The binding free energy (ΔG_bind_) is calculated considering a thermodynamic cycle that constitute the following terms:


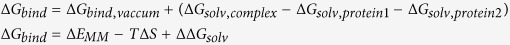


where the average interaction energy between two proteins in vacuum (ΔE_MM_) is calculated as molecular mechanical energy, entropy change upon binding (TΔS) is estimated from normal mode analysis if necessary, and the change in solvation free energy (ΔΔG_solv_) is estimated by solving the Generalized Born equation for each of the protein-protein complex and both unbound proteins, and adding an empirical term for hydrophobic contributions to it. The hydrophobic contribution is calculated from the solvent accessible surface area (SASA). Entropic calculation has been omitted here for simplicity. The interactions between different segments of p27 and CDK2 were obtained by residue-level decomposition of the protein-protein binding free energy. The reported average ΔG_bind_ was obtained from five independent windows of 2 ns each from the last 10 ns trajectory.

## Additional Information

**How to cite this article**: Rath, S. L. and Senapati, S. Mechanism of p27 Unfolding for CDK2 Reactivation. *Sci. Rep*. **6**, 26450; doi: 10.1038/srep26450 (2016).

## Supplementary Material

Supplementary Information

Supplementary Movie S1

## Figures and Tables

**Table 1:** Binding free energy of ATP to CDK2 catalytic cleft in various CDK2/CyclinA complexes.

Complex No.	Complex	ΔG_binding_ (kcal/mol)
1	Crystal structure of active CDK2/CyclinA	−6.59 ± 0.52
2	pY88-p27/CDK2/CyclinA	−6.16 ± 0.41
3	p27/CDK2/CyclinA	+163.71 ± 20.14

Results are obtained from protein-ligand docking studies using AutoDock. The receptor’s flexibility has been introduced in the calculations through Relaxed Complex Scheme (ref. 29). The ATP-bound active CDK2/CyclinA crystal structure (complex 1) has been used to standarise the docking protocol, in which the autodock run parameters were iterated to produce ΔG_binding_ value similar to the experimental value of −6.30 kcal/mol (ref. 30) for the known pose of ATP binding to the crystal structure. The same parameters were subsequently used for the test cases, complex 2 and 3.
