# Supplementary material for: Mechanism of p27 Unfolding for CDK2 Reactivation
Source: Sci Rep. 2016 May 23;6:26450. doi: 10.1038/srep26450 (PMC4876385; doi:10.1038/srep26450)
Supplement: Supplementary Information [file srep26450-s1.pdf]

Mechanism of p27 Unfolding for CDK2 Reactivation

Soumya Lipsa Rath and Sanjib Senapati

Table S1. List of systems studied

Serial No.	System	Technique Used	Time
1	p27/CDK2/CyclinA	cMD	200ns
2	pY88p27/CDK2/CyclinA	cMD	200ns
3	p27/CDK2/CyclinA	aMD	200ns
4	pY88p27/CDK2/CyclinA	aMD	200ns

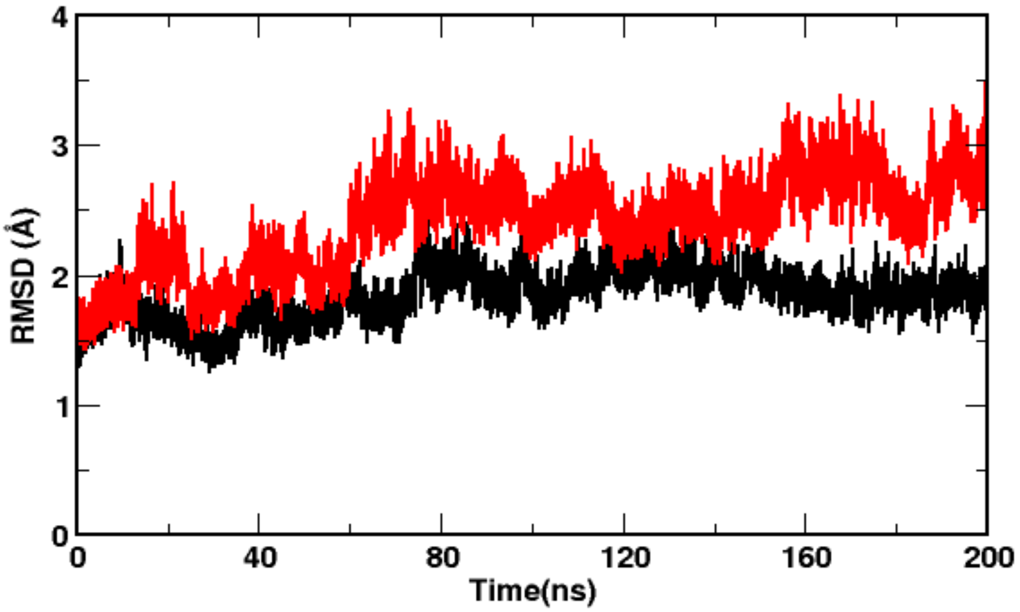

Fig. S1 Time evolution of the RMSD of p27/CDK2/Cyclin A complex during classical MD simulation. RMSD of unphosphorylated (black) and phosphorylated (red) ternary complex is shown. RMSDs are calculated with respect to the C_α atoms of the protein residues.

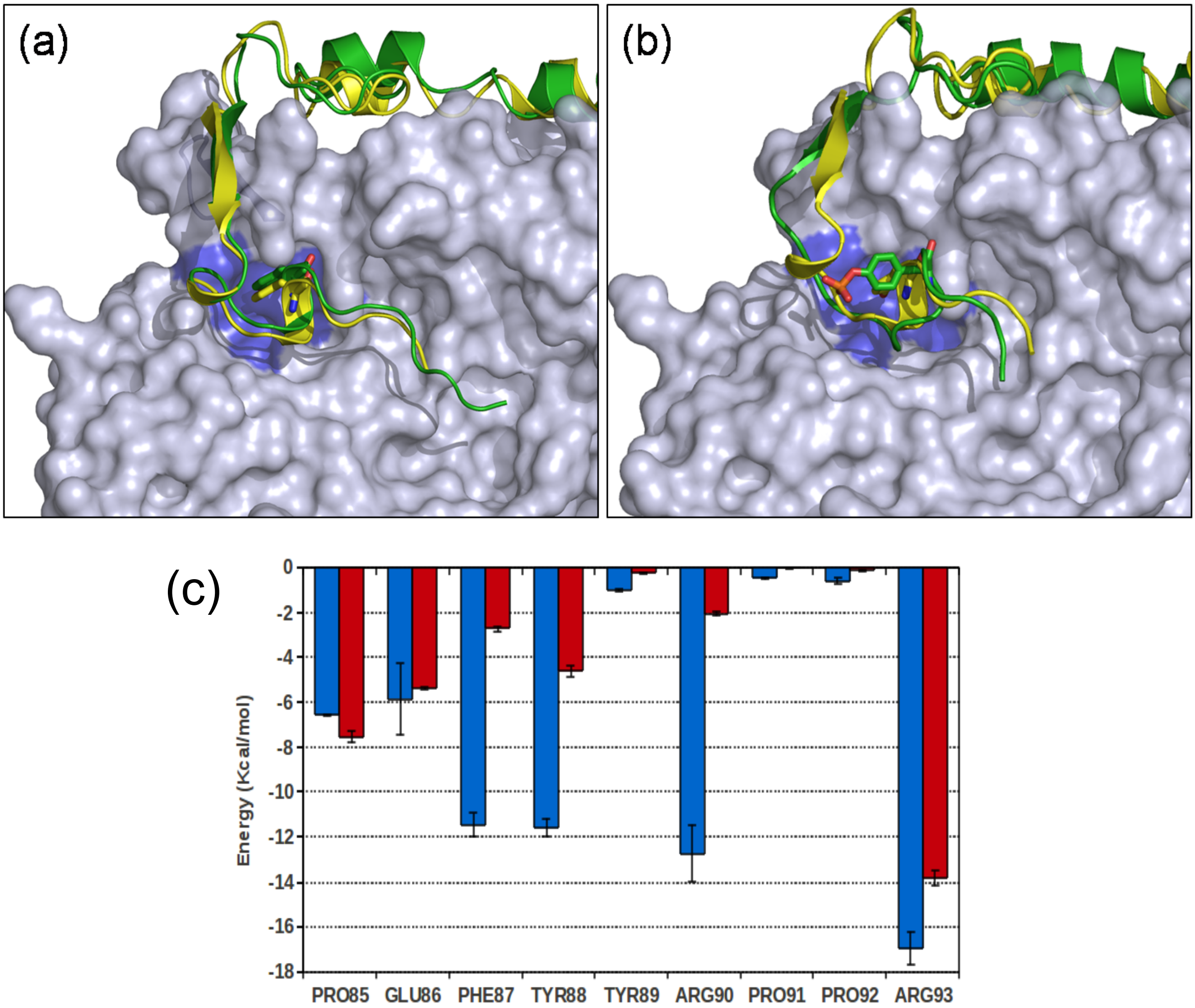

Fig. S2 Structures of p27 from classical MD simulation. Time-averaged conformations of p27 (green) and CDK2/Cyclin A (ice blue) in (a) p27/CDK2/Cyclin A and (b) pY88-p27/CDK2/Cyclin A complexes from cMD simulations. For comparison, the p27 conformations from simulations are superposed onto the crystal structure in yellow. Phosphorylated Y88 in p27 is shown in sticks, with P in orange and O in reds. CDK2 catalytic pocket is highlighted in blue. (c) Interaction energy of p27 3_{10}-helix residues with CDK2 catalytic cleft in unphosphorylated (blue) and phosphorylated (red) complexes. Total computed interaction energy between p27 and CDK2 in the simulated complexes (a) and (b) was -143.89 kcal/mol and -142.67 kcal/mol, respectively. Energy values were computed by Molecular Mechanics Generalized Born surface area (MMGBSA) method, using the AMBER tool.

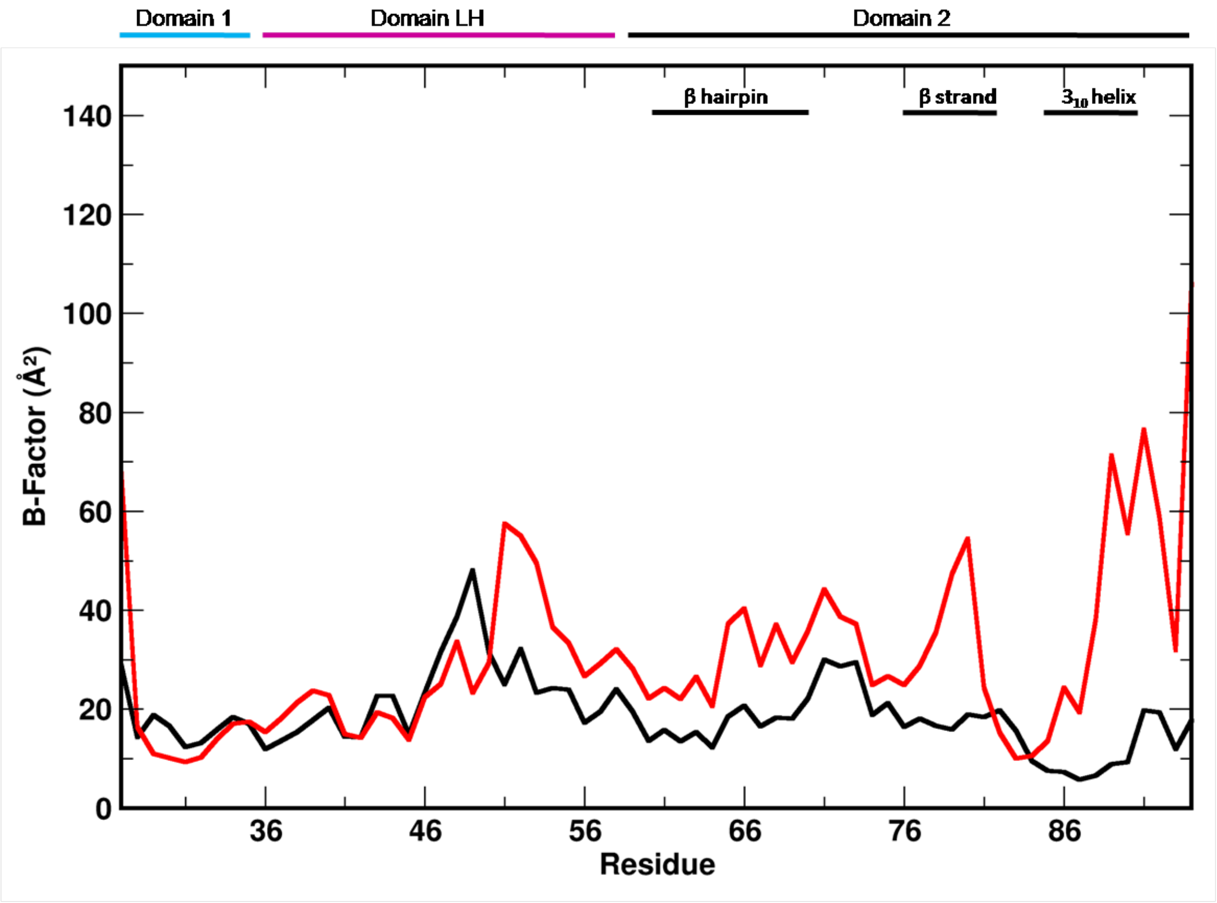

Fig. S3 Fluctuations in p27 residues. Comparison of the B-factors of C_α atoms of p27 in unphosphorylated and phosphorylated complexes from cMD simulations. Colour scheme is similar to S1 Fig.

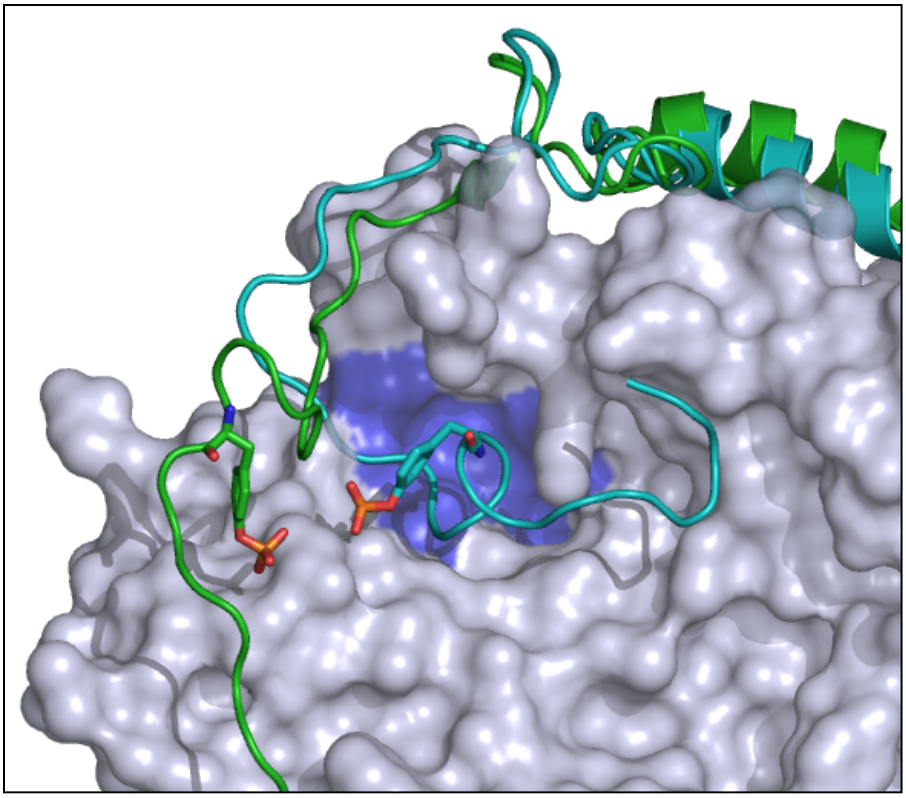

Fig. S4 Conformational changes in p27 from accelerated MD simulation. Representative conformations of p27 from the two distinct ensemble of structures in Fig 2d (aMD simulation of phosphorylated complex). Cyan represents a conformation where RMSD of p27 is ~5 A, which corresponds to a p27 bound state to CDK2/cyclinA. Green represents a conformation where RMSD of p27 is ~ 11 A, which corresponds to p27 unbinding. Phosphorylated Y88 in p27 is shown in sticks, with P in orange and O in reds. CDK2 catalytic pocket is highlighted in blue.

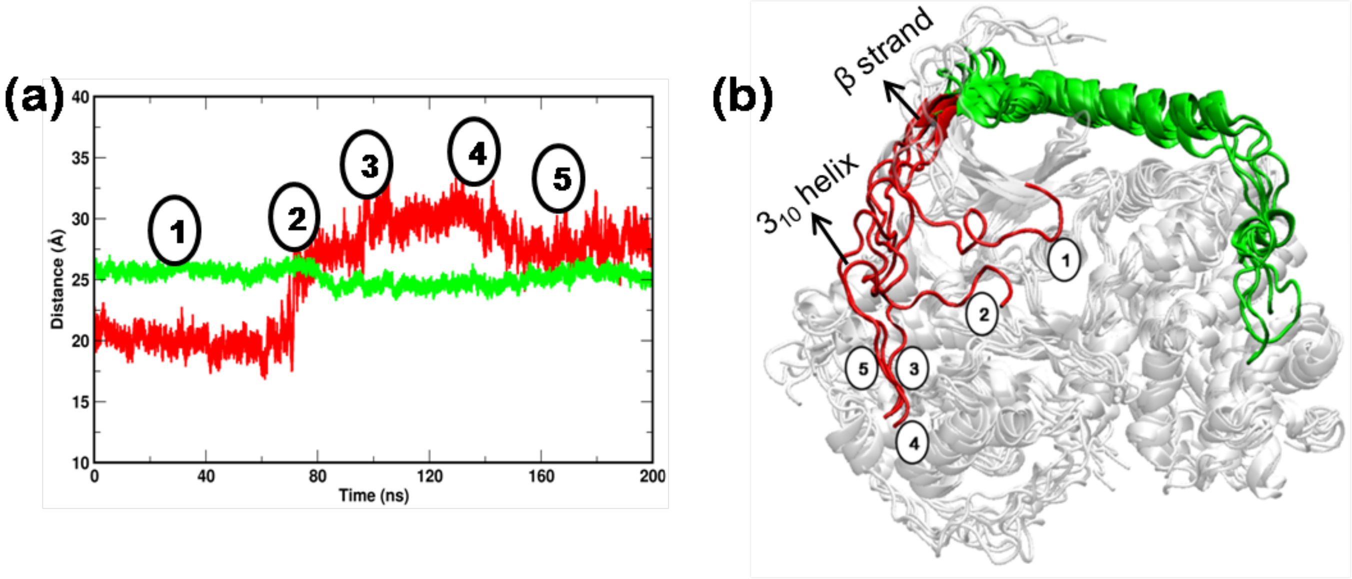

Fig. S5 Time evolution of the detachment of p27 from CDK2. (a) Time evolution of the distance of p27 β-strand plus 3_{10} helix (red) and rest of the p27 structure (green) from the centre of mass of CDK2/Cyclin A complex. The long-lived conformations of β-strand and 3_{10} helix are marked by numbers. (b) The structure of p27 at the corresponding long-lived conformations. Superposition of CDK2/Cyclin A complex (white) at various time shows a minute change in the conformation of CDK2 and Cyclin A.

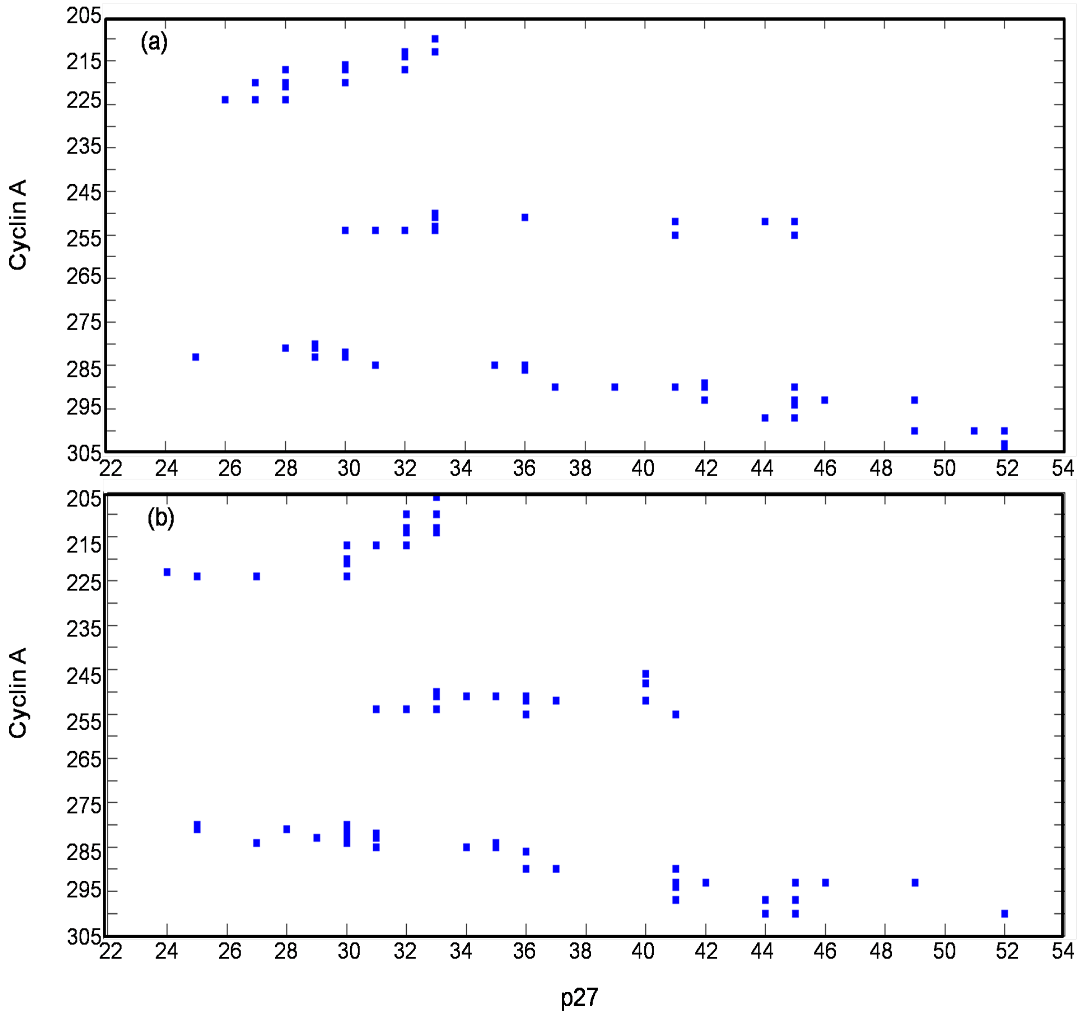

Fig. S6 Comparison of the p27/Cyclin A interfacial contacts. Interfacial contacts are shown for (a) unphosphorylated and (b) phosphorylated complexes from aMD simulations. Each square represents contact between a pair of residues.

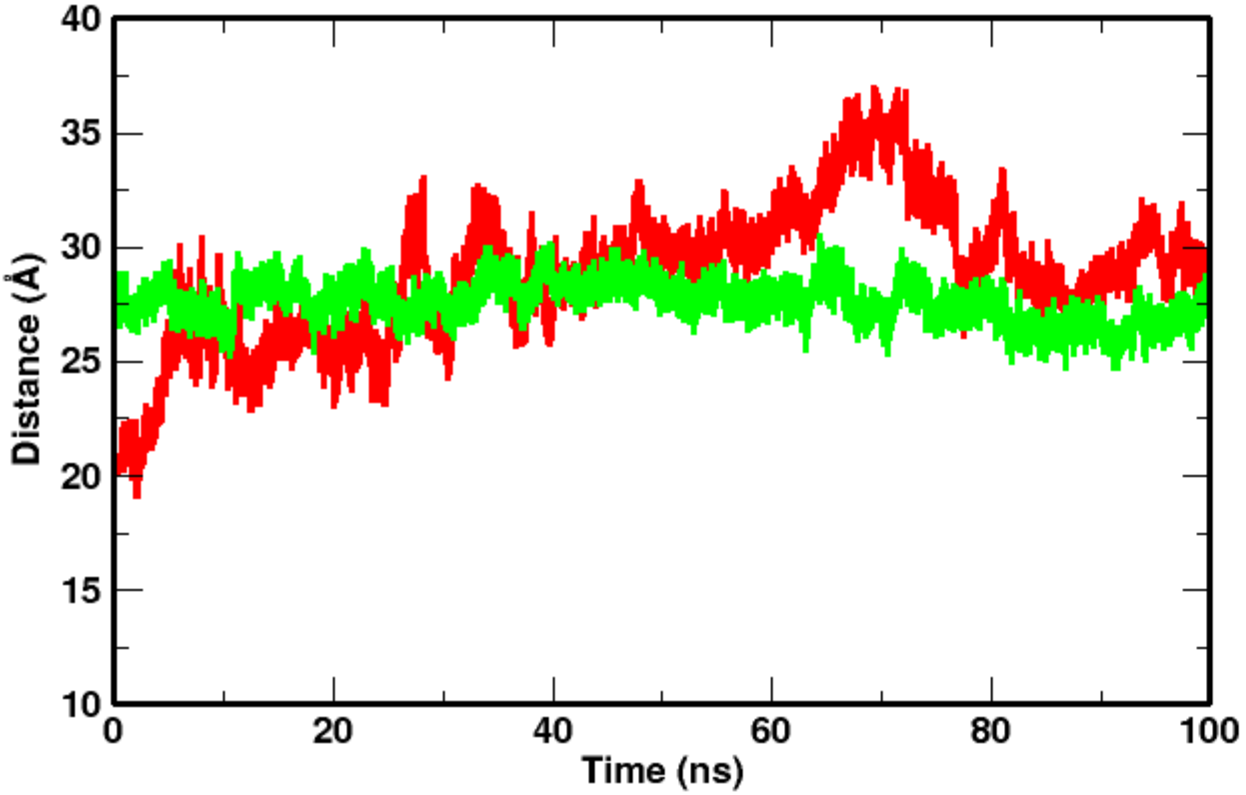

Fig. S7 Results from *in silico* mutagenesis study. Time evolution of the distance of p27 β-strand plus 3_{10} helix (red) and rest of the p27 structure (green) from the centre of mass of CDK2/Cyclin A complex in the mutational study of p27:K81A. Results are shown from the 100 ns phosphorylated aMD simulation. The breaking of hybrid β-sheet and 3_{10} helix took place in 45ns compared to 80ns in WT (see S5a Fig.).

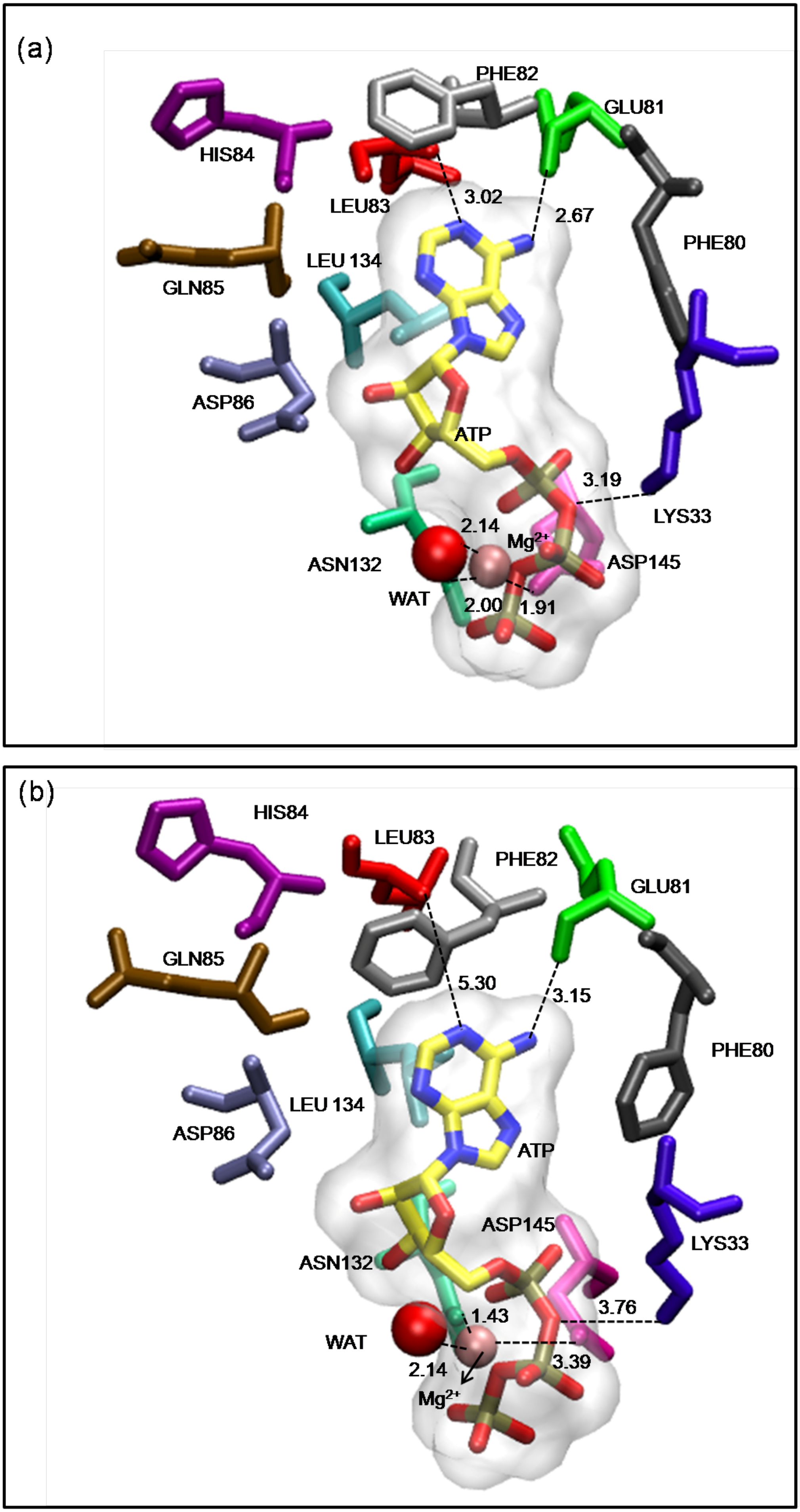
(a)
PHE82
GLU81
HIS84
LEU83
3.02
2.67
PHE80
GLN85
LEU 134
ASP86
ATP
3.19
LYS33
2.14
Mg^{2+}
ASN132
ASP145
WAT
2.00
1.91
(b)
HIS84
LEU83
PHE82
GLU81
GLN85
5.30
3.15
PHE80
LEU 134
ASP86
ATP
ASP145
LYS33
ASN132
1.43
3.76
WAT
3.39
2.14
Mg^{2+}

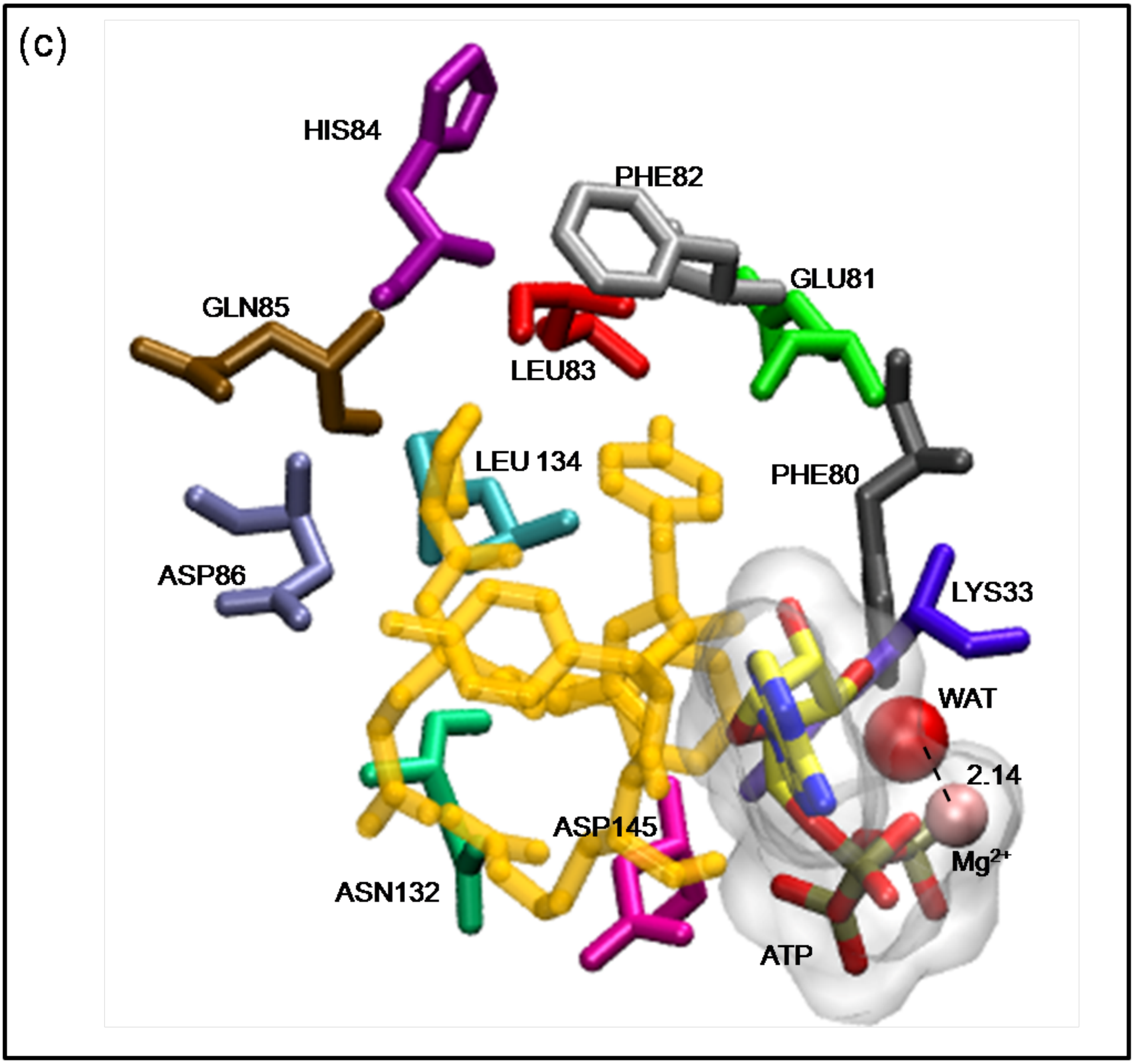

Fig. S8 Comparison of ATP binding to the CDK2 catalytic pocket in different CDK2/CyclinA structures. Results are shown for the ATP binding pose in (a) crystal structure of active CDK2/ CyclinA (PDB ID: 1QMZ), (b) time-averaged structure of phospho-p27/CDK2/CyclinA from aMD simulation and (c) crystal structure of p27/CDK2/CyclinA. The structure of CDK2 in (b) and (c) are superposed on the CDK2 structure in (a) for structural alignment of the protein complexes. The Mg^{++} ion and crystal water as found in the crystal structure of active CDK2/CyclinA complex in (a) were included in the docking studies for the complexes in (b) and (c). Important contacts between ATP and CDK2 residues are shown. CDK2 residues are shown in stick representation and Mg^{++} ion and crystal water are shown in sphere. ATP is coloured by name with 'C' in yellow, 'N' in blue, 'O' in red and 'P' in bronze. In (c), presence of p27 is shown in orange. Hydrogens are omitted for clarity. CDK2-ATP distances are calculated through heavy atoms: LEU83N-ATP, GLU81OE2-ATP, ASN132OD1-Mg^{++}, ASP145OD2-Mg^{++} and LYS33NZ-ATP and shown by dotted lines.

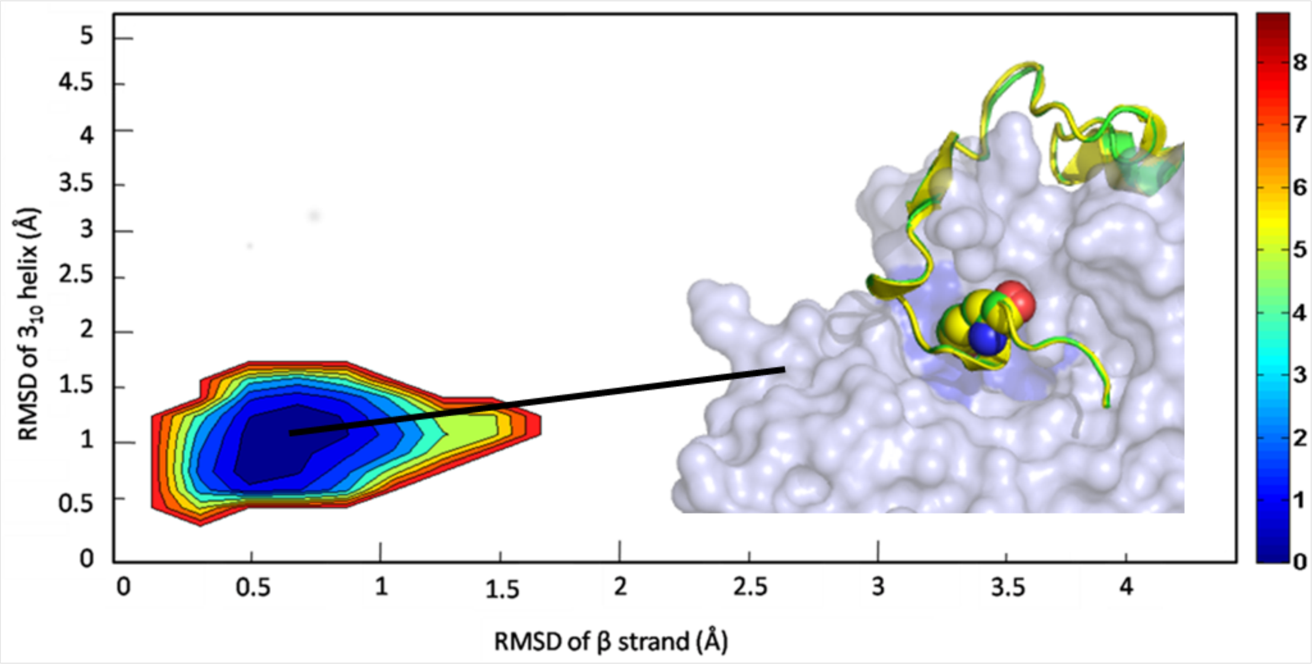

Fig. S9 Free energy profile of unphosphorylated p27. Free energy landscape of p27 from the aMD simulation of unphosphorylated p27/CDK2/Cyclin A complex. In inset, the most populated structure of p27 (green) is superposed with its structure in crystal (yellow). Unphopsphorylated 3_{10} helix of p27 could not exit from the substrate binding pocked of CDK2, even after 200ns of aMD simulation.

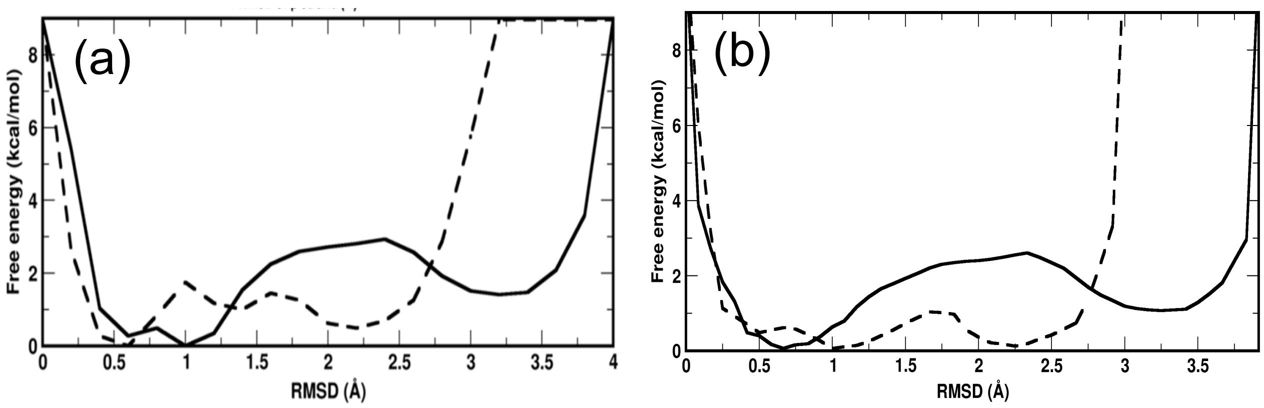

Fig. S10 Comparison of the free energy profiles using different reweighting algorithms. Comparison of the free-energy profile of p27 unbinding using (a) Boltzmann reweighting and (b) Maclaurin series expansion reweighting algorithms.

Movie. S1 The mechanism of p27 unbinding from CDK2/CyclinA complex. The unbinding process is initiated by the loss of p27-CDK2 hybrid β-sheet, which is followed by the ejection of 3_{10} helix from the CDK2 catalytic pocket (shown in green). The relocation of the crystal-structure-missing CDK2 β1-strand and G-loop to their functional positions is also shown (in magenta). CDK2/Cyclin A backbone is shown in opaque white.
